# Intestinal Microbiota in Healthy U.S. Young Children and Adults—A High Throughput Microarray Analysis

**DOI:** 10.1371/journal.pone.0064315

**Published:** 2013-05-23

**Authors:** Tamar Ringel-Kulka, Jing Cheng, Yehuda Ringel, Jarkko Salojärvi, Ian Carroll, Airi Palva, Willem M. de Vos, Reetta Satokari

**Affiliations:** 1 Department of Maternal and Child Health, Gillings School of Global Public Health, The University of North Carolina at Chapel Hill, Chapel Hill, North Carolina, United States of America; 2 Department of Veterinary Biosciences, Division of Microbiology and Epidemiology, University of Helsinki, Helsinki, Finland; 3 Division of Gastroenterology and Hepatology, Department of Medicine, The University of North Carolina at Chapel Hill, Chapel Hill, North Carolina, United States of America; 4 Haartman Institute, University of Helsinki, Helsinki, Finland; 5 Laboratory of Microbiology, Wageningen University, Wageningen, The Netherlands; University of Florida, United States of America

## Abstract

It is generally believed that the infant's microbiota is established during the first 1–2 years of life. However, there is scarce data on its characterization and its comparison to the adult-like microbiota in consecutive years.

**Aim:**

To characterize and compare the intestinal microbiota in healthy young children (1–4 years) and healthy adults from the North Carolina region in the U.S. using high-throughput bacterial phylogenetic microarray analysis.

**Methods:**

Detailed characterization and comparison of the intestinal microbiota of healthy children aged 1–4 years old (n = 28) and healthy adults of 21–60 years (n = 23) was carried out using the Human Intestinal Tract Chip (HITChip) phylogenetic microarray targeting the V1 and V6 regions of 16S rRNA and quantitative PCR.

**Results:**

The HITChip microarray data indicate that Actinobacteria, Bacilli, Clostridium cluster IV and Bacteroidetes are the predominant phylum-like groups that exhibit differences between young children and adults. The phylum-like group Clostridium cluster XIVa was equally predominant in young children and adults and is thus considered to be established at an early age. The genus-like level show significant 3.6 fold (higher or lower) differences in the abundance of 26 genera between young children and adults. Young U.S. children have a significantly 3.5-fold higher abundance of *Bifidobacterium* species than the adults from the same location. However, the microbiota of young children is less diverse than that of adults.

**Conclusions:**

We show that the establishment of an adult-like intestinal microbiota occurs at a later age than previously reported. Characterizing the microbiota and its development in the early years of life may help identify ‘windows of opportunity’ for interventional strategies that may promote health and prevent or mitigate disease processes.

## Introduction

The intestinal microbiota has a significant role in human health. Changes in the composition of the intestinal microbiota have been observed in relation to various gastrointestinal (GI) [1], [2], [3], [4], [5], [6], [7], [8], [9] and non- GI[10], [11], [12], [13], [14], [15] disease conditions both in children and adults. Accumulating evidence on the dysbiosis of the intestinal microbiota (i.e., changes in the composition and/or function of the intestinal microbiota) associated with disease conditions has led to increased interest in interventions targeting this complex microbial community (e.g., prebiotics, probiotics and antibiotics) aiming to improve or restore the ‘normal’ microbiota, as means for maintaining and/or improving health, preventing and/or treating diseases[16], [17].

Specifically, with regard to children, the emerging data suggest that compositional changes in the intestinal microbiota in early life may lead to development of disease conditions at a later age. Epidemiological investigations have shown that the use of antibiotics early in life is associated with increased risk for asthma[18], [19] and Crohn's disease[20] later in childhood and adulthood. Microbiological investigations have shown that reduced intestinal bacterial diversity in infancy is associated with a higher risk for allergic rihinitis at 6 years of age[21] and that lower levels of *Bifidobacteria* in young children are associated with overweight at 7 years of age [10], [22].

The development, establishment, and dynamic changes of the human intestinal microbiota during life are not well-understood. Many factors can affect the acquisition, succession, and colonization of the infant's gut including mode of delivery[23], [24], breastfeeding vs. formula feeding [24], [25], and genetics [26]. Although numerous studies have investigated the composition of the intestinal microbiota in infants and adults, it is currently unknown at what age the infant's microbiota is established and reaches an adult-like profile [27], [28], [29], [30], [31]. Most of the research on the intestinal microbiota in children has focused on characterizing the microbiota in the first years of life and there is very limited data on the intestinal microbiota of toddlers and older children. Furthermore, until recently, most of the reported data on the intestinal microbiota in this age group were based on studies using conventional techniques (e.g., culture, qPCR and *in situ* hybridization). In a recent study by Yatsunenko et al., a series of advanced high-throughput techniques, including deep Illumina sequencing of 16S rRNA gene fragments and metagenome analysis, were applied to over 500 subjects from 3 continents, including nearly 100 children below 3 years of age. This enabled a comprehensive, in depth characterization of the intestinal microbiota and functional genes across age and populations [32]. The study highlighted the commonalities and differences through maturation between populations and cultures as the subjects originated from rural Malawi, the Amazone region, and metropolitan areas in the U.S. and emphasized the importance of further research to characterize the gut microbiome between different sub-populations/regions within specific populations.

The aim of this study was to characterize the composition of the intestinal microbiota in 51 subjects from a single geographical region within the U.S. and specifically compare the microbiome from healthy young children aged 12–48 months with that of healthy adults aged 21–65 years, using high-throughput bacterial phylogenetic microarray analysis (HITChip).

## Methods

### Study Population

We studied fecal samples collected from healthy children 12–48 months of age (n = 28) and healthy adult subjects 21–60 years of age (n = 23). Adult subjects were recruited from the general population by advertising and children were recruited from twenty nine child care centers in three counties in the Piedmont area of North Carolina, U.S. Our study concentrated on a relatively homogeneous population in a specific geographical region of one state in which the diet is anticipated to be Western diet with relatively few deviations. All children were attending child-care-centers which have some consistency of diet with respect to the broad nutritional requirements, per local state and federal rules and regulations.

Potential study subjects were selected for study participation if they met the following criteria: 1–4 years of age for the children group, 18 years of age or older for the adult group, no history of treatment with antibiotics within the previous 4 weeks, and no intentional consumption of probiotics within two weeks prior to study participation. Subjects were of any gender, BMI (body mass index), or ethnicity and had no acute or chronic illness or symptoms. Body mass index (BMI) was calculated per standard definition (kg/m^2^). For adults, weight and height were collected at their visit and BMI was calculated and characterized as healthy weight (BMI 18.5–24.9), overweight (BMI 25–29.9) and obese (BMI ≥30). For children, weight and height were collected at the child care center by trained research assistants. BMI percentile was determind using the World Health Organization (WHO) age and sex adjusted reference standards. Applying these percentiles, children were characterized as healthy (5–84.9%), overweight (85%–94.9%) or obese (≥95%). The demographics characteristics of these study subjects are compiled in Table S1. The study was approved by the UNC Internal Review Board (IRB) and all adult subjects and parents/guardians of children provided written consent prior to study participation.

### Sample Collection and Preparation

Fresh stool samples were collected from adult subjects on site when possible or in the morning of a single study visit at UNC as previously described [6], [33]. For children, parents/guardians were instructed to collect a fresh fecal sample and return it to study staff at the child care center on the same morning in a small cooler with an ice pack provided by the study. At the center, samples were transferred to a larger cooler with ice packs for transport to UNC. Fecal sample collection and handling were similar for both children and adults. Each fecal sample was transferred to the laboratory in a cooler (−4°C) where it was mechanically homogenized with a sterile spatula, divided into aliquots, and stored at −80°C for future DNA isolation and molecular microbiological analysis.

### Isolation of DNA

DNA was isolated from fecal samples using a phenol/chloroform extraction method combined with physical disruption of bacterial cells and a DNA clean-up kit (Qiagen DNeasy® Blood and Tissue extraction kit, Qiagen, Valencia, CA) as previously described[6]. Isolated fecal DNA was quantified using a nanodrop (Thermo Scientific, Asheville, NC) before being used for subsequent microbiological analysis.

### Phylogenetic Microarray analysis

The analysis of fecal DNA has been described previously [34], [35]. In short, the 16S rRNA gene was amplified from 40 ng of DNA as template with primers T7 prom-Bact-27-for and Uni-1492-rev, followed by *in vitro* transcription and labeled with Cy3 and Cy5 in order to carry out duplicate runs. All procedures have been described previously in more detail by Biagi et al. and Rajilic' et al [34], [35]. The mixtures of labeled RNA samples were fragmented before hybridizing in each array [34], [35]. Human Intestinal Tract Chip (HITChip) arrays were scanned by the Agilent Microarray Scanner G2505C (Agilent, USA). Intensity values for each image were extracted with the Agilent Feature Extraction software, version 10.7.3.1. Normalization and quality control of HITChip array data was performed with scripts in R statistical software, as described by Rajilic et al. and Salonen et al. [35], [36]. The between sample normalization was carried out with min-max algorithm [37], [38].

The HITChip bacterial phylogenetic microarrays consist of 4800 oligonucleotide probes targeting the V1 and V6 hypervariable regions of the 16S rRNA gene and covering over 1,033 intestinal phylotypes or bacterial species [34], [39], [40]. The HITChip microarray allows a comprehensive and high-resolution analysis of the microbiota composition in several taxonomic levels from phylotypes (or bacterial species) to genus-like level (L2) and phylum-like level (L1). The duplicate runs of all samples indicated strong agreement (Pearson's correlation over 95%).

### Statistical analysis

The HITChip data analysis was performed in R version 2.12.1 [41], and focused on 22 phylum-like and 130 genus-like phylogenetic groups on the array. Genus-like taxa correspond to bacteria having 90% or higher similarity in their 16S rRNA gene [35], [40], and phylum-like taxa, correspond to a phylum or to the Clostridium clusters within Firmicutes. The sum of signal intensities for probes targeting a genus-like (or phylum-like) group was used as quantitative measure of the abundance of that group in the sample [39]. When computing the signal on higher-level taxa, the probe intensities were divided by the number of known target phylotypes per probe to insure that the total intensity in each level corresponded to the actual total intensity.

The diversity of the microbial profiles was assessed by computing the Simpson's reciprocal index of diversity [42] and Shannon index of diversity [43] from oligo level data. The significances of the diversities between age groups were assessed by Student's t-test and adjusted by Benjamini-Hochberg (BH) false discovery rate (FDR) correction. Principal Component Analysis (PCA) and Redundancy Analysis (RDA) [44] were computed using R packages stats and vegan [44], [45]. The significance of separation in RDA analysis was estimated with a permutation test [44] using 5,000 permutations. The multiple comparisons between BMI categories on microbiota composition were carried out with linear models in R using the packages stats and multcomp [46]. Similarity of microbiota compositions was estimated with Pearson's correlation between samples, and visualized with hierarchical clustering using complete linkage algorithm [47]. For each taxon, the statistical significance of separation between groups was estimated with Student's t-test assuming equal variance and two- tailed distribution. FDR correction of p-values was then carried out with BH method [48]. The relative contribution of each genus-like phylogenetic group was calculated by first computing the percentage of signal intensity within each sample, and then computing the averages of relative abundances in adults and children groups. The significance of the average relative abundance of each genus-like taxa between adults and children was estimated with Student's t-test and corrected by BH. For all analyses, FDR-corrected p-values below 0.05 were considered significant.

The statistical significance of the effect of gender, BMI category and ethnicity on microbial compostions within the children or adults groups was tested using linear models in R.

### Quantitative PCR

The amount of *Bifidobacterium* species in children and adult samples were verified using quantitative real-time PCR (qPCR). qPCR was performed using the SYBR® Green PCR master mix (Applied Biosystems, Carlsbad, CA) with primers that amplify the genes encoding 16S rRNA from *Bifidobacterium* genus [49] (forward, 5′GGGTGGTAATGCCGGATG-3′; reverse, 5′- TAAGCGATGGACTTTCACACC-3′) [6]. The numbers of the genus *Bifidobacterium* was determined in all fecal samples as copies of the 16S rRNA gene per µg of fecal DNA. The levels of *Bifidobacterium* species were compared between groups using the Student's *t* test.

## Results

### I. Study Population and HITChip general data

A total of 51 subjects were investigated. The children (n = 28) had a median age of 2.7 years (range 1–4). The child population consisted of 46% (n = 13) males and 54% (n = 15) females, 59% (n = 16) white, 26% (n = 7) black, 11% (n = 3) hispanic, 3.7% (n = 1) pacific-islander and one child's ethnicity was not recorded. The adult study population (n = 23) were 21–60 years of age with a median of 28 years, 83% (n = 19) females, 87% (n = 20) whites, 9% (n = 2) black and 4% (n = 1) asian. HITChip analysis detected 1,038 species-like groups, 130 genus-like groups and 22-phylum-like groups in the fecal samples of the studied population (children and adults). The demographics information of study subjects are compiled in Table S1 and Figure S1. Among children, gender, ethnicity, BMI category or age group had no significant effect on microbiota profile as is shown in PCA and RDA analysis (Figure S2). In adults, gender, ethnicity and BMIcategories were heavily biased in distribution (Table S1, Figure S1). Nevertheless, the possible effect of these confounding factors on the microbiota composition was assessed and no significant effect on the total microbiota profile was found (Figure S3). Further, in order to assess in more detail the possible effect of gender, ethnicity or BMI category on microbiota composition we applied linear model on the genus-like level HITChip data. In the children's group, none of the bacterial groups was found to be affected by gender, ethnicity or BMI category (data not shown). In the adults group, one bacterial group was affected by gender, six by ethnicity and one by BMI category (data not shown). None of these bacterial groups were among those showing significant difference between children and adults (reported below).

### II. Characterization of the intestinal microbiota at phylum-like (L1) and genus-like (L2) levels

#### A. Principal component analysis

**Figure 1:**
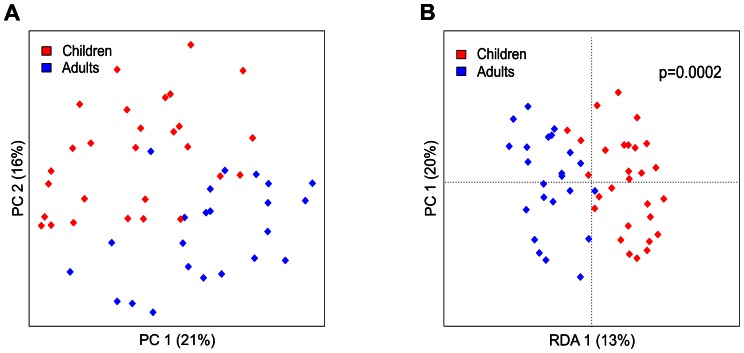
Principal component analysis (PCA) (A) and redundancy analysis (RDA) (B) of fecal samples from healthy young children and adults at the phylum-like level. Log transformed data were used for analysis. In PCA, The first two principal components capture 21% (PCA1) and 16% (PCA2) of variance respectively. RDA plot shows the result from supervised PCA, where group assignment of subjects (adults or children) was used as a dependent variable. In RDA, first and second ordination axes are plotted, explaining 13% and 20% of the variance. Separation between children and adults is significant (p = 0.0002, permutation test).

**Figure 2:**
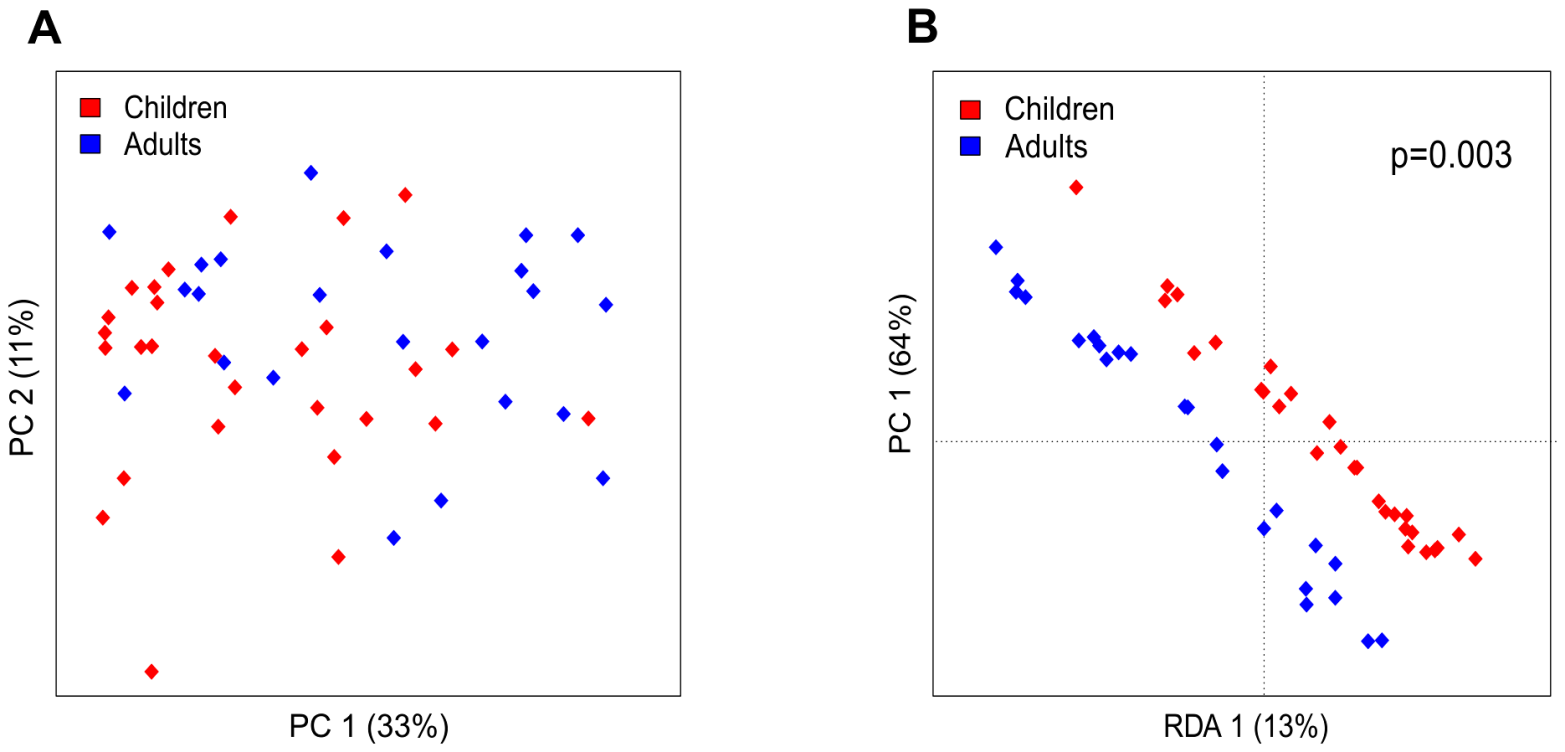
Principal component analysis (PCA) (A) and redundancy analysis (RDA) (B) of fecal samples from healthy young children and adults at the genus-like level. Log transformed data were used for analysis. In PCA, the first two principal components capture 33% (PCA1) and 11% (PCA2) of variance respectively. RDA plot shows the result from supervised PCA, where group assignment of subjects (adults or children) was used as a dependent variable. In RDA, first and second ordination axes are plotted, explaing 13% and 64% of the variance. Separation between children and adults is significant (p = 0.003, permutation test).

The HITChip microarray profiling revealed significant differences in the total microbiota composition between healthy young children and adults. Principal Component Analysis (PCA) and Redundancy Analysis (RDA) separated young children and adults based on the phylum-level microbiota composition. The first principal component (PC) revealed separation that explained 21% of variance while the second PC explained 16% of variance (Figure 1). Separation between adults and children was statistically significant in RDA analysis (p = 0.0002). Gender, ethnicity or BMI category did not separate groups based on the phylum-level microbiota profiles (Figure S4). In the PCA of the genus-like level microbiota profiles children and adults did not cluster separately (Figure 2). However, the RDA of the genus-like level microbiota profiles separated young children from adults (p = 0.003) (Figure 2). In total, 26 genus-like bacterial groups with relative abundance of at least 0.01% of the total bacterial population illustrated significant differences between adults and young children (Table 1).

**Table 1:** Comparison of bacterial groups showing significant differences in fecal samples from adults and healthy young children.*

		Relative contribution (%)^A^		Ratio^B^	p-value^C^
Phylum/order	Genus-like phylogenetic group	Adults	Children	A/C	A vs. C
Actinobacteria	*Bifidobacterium*	2.893	10.958	0.264	8E-05
Bacilli	*Lactobacillus plantarum et rel.*	0.045	0.072	0.625	0.019
	*Streptococcus bovis et rel.*	0.672	2.617	0.257	0.014
	*Streptococcus intermedius et rel.*	0.127	0.289	0.439	0.039
	*Streptococcus mitis et rel.*	0.409	1.267	0.323	0.019
Bacteroidetes	*Alistipes et rel.*	1.482	0.365	4.066	0.019
	*Bacteroides intestinalis et rel.*	0.525	0.159	3.295	0.021
	*Bacteroides splanchnicus et rel.*	0.808	0.268	3.015	0.032
	*Bacteroides stercoris et rel.*	0.956	0.151	6.323	0.032
	*Bacteroides vulgatus et rel.*	1.609	0.580	2.772	0.029
	*Prevotella tannerae et rel.*	0.808	0.287	2.817	0.028
	*Tannerella et rel.*	0.531	0.175	3.027	0.019
	*Uncultured Bacteroidetes*	0.011	0.005	2.380	0.029
Clostridium cluster III	*Clostridium stercorarium et rel.*	1.963	0.511	3.841	0.039
Clostridium cluster IV	*Eubacterium siraeum et rel.*	0.517	0.080	6.465	0.045
	*Oscillospira guillermondii et rel.*	3.582	0.738	4.855	0.037
	*Sporobacter termitidis et rel.*	0.883	0.327	2.699	0.049
Clostridium cluster XIII	*Peptostreptococcus micros et rel.*	0.020	0.031	0.670	0.028
Clostridium cluster XIVa	*Butyrivibrio crossotus et rel.*	0.902	0.441	2.045	0.012
Fusobacteria	*Fusobacteria*	0.016	0.023	0.667	0.028
Proteobacteria	*Campylobacter*	0.018	0.026	0.706	0.045
	*Helicobacter*	0.012	0.017	0.670	0.028
	*Klebsiella pneumoniae et rel.*	0.013	0.020	0.655	0.045
	*Proteus et rel.*	0.011	0.016	0.677	0.029
	*Vibrio*	0.012	0.017	0.695	0.032
	*Yersinia et rel.*	0.010	0.016	0.613	0.011

* Table includes groups at the genus-like level that constitute 0.01% or more of the total signal (total microbiota) in both adults and

young children, and show significant difference between the study groups.

A Relative contribution of genus-like phylogenetic group is the average of relative abundances of the subjects in adults or children groups.

B Ratio of the average relative abundance for each genus-like phylogenetic group. A – adults and C – children.

C Adjusted p values from two sample t-test followed with the correction by Benjamini-Hochberg false discovery rate correction (p<0.05 significant).

#### B. Taxa abundance

**Figure 3:**
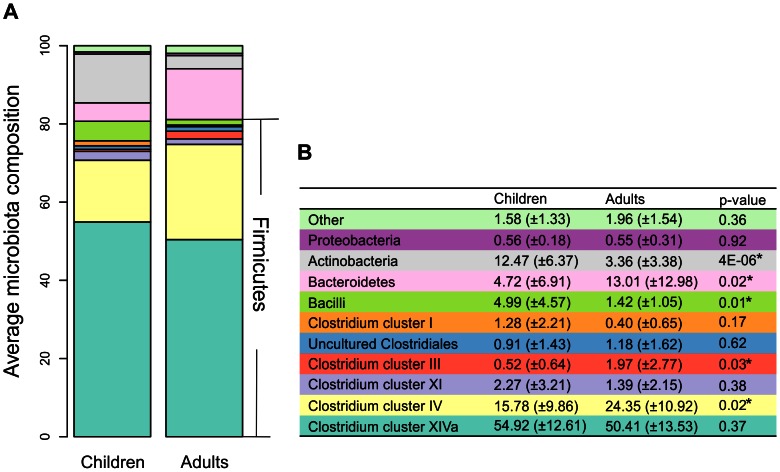
Phylum-level distribution of bacteria in fecal samples of healthy young children and adults.

At the phylum level, we found that the most predominant phyla both in young children (C) and adults (A) are Firmicutes (mostly Clostridium clusters, C:80.67%, A:81.12%), Bacteroidetes (C:5%, A:13%) and Actinobacteria (C:12.5%, A:3.4%) (Figure 3a). Five phylum-like groups showed significant differences between young children and adults (Figure 3b). There was no difference in the mean Firmicutes to Bacteroidetes ratio between young children and adults (64 and 48 respectively; p = 0.36).

**Figure 4:**
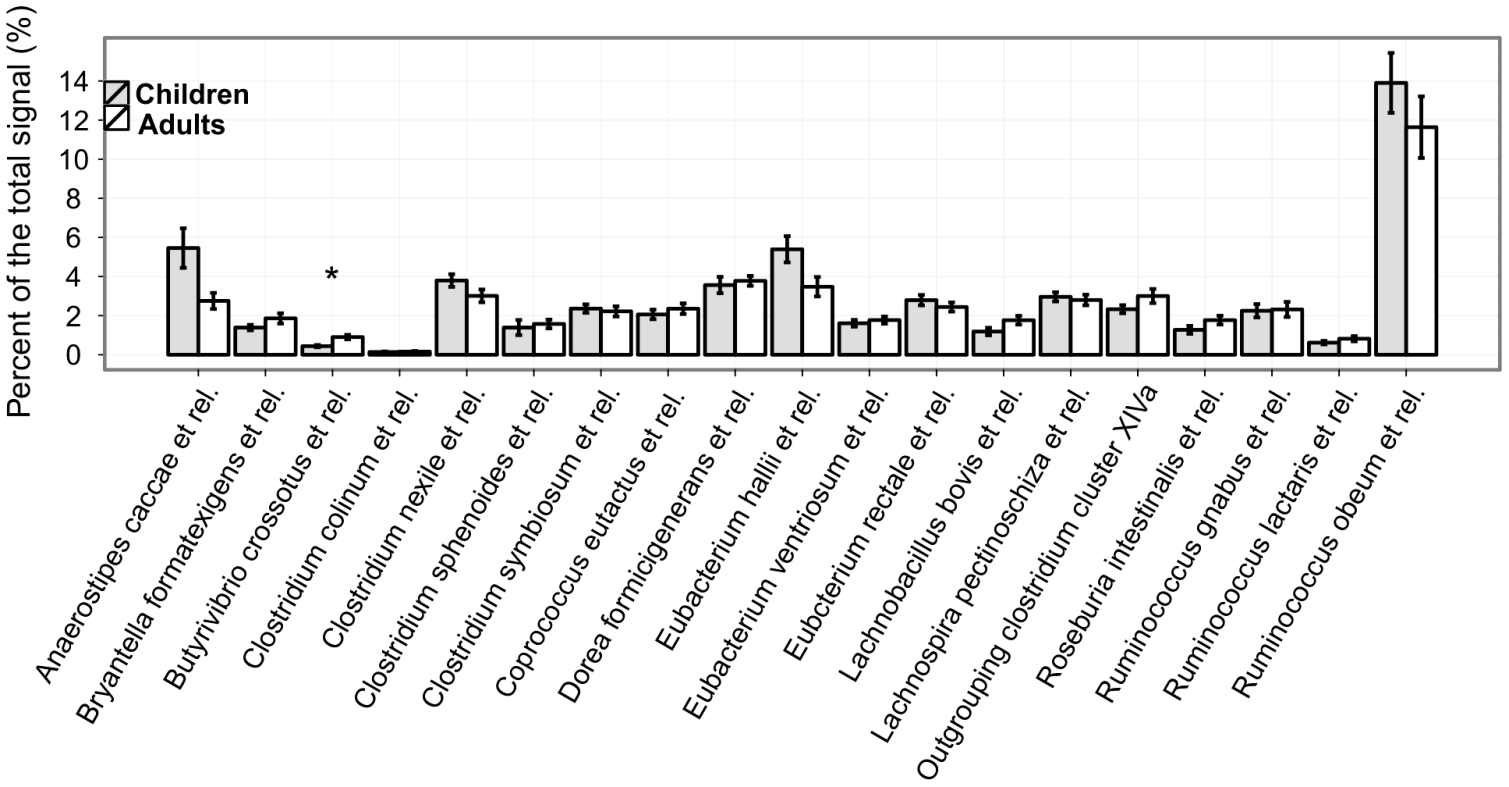
Genera within the Clostridium cluster XIVa. The error bar shows the standard error.

Clostridium cluster XIVa was the most predominant taxon in both groups and was equally abundant in adults and young children (50.4% vs. 54.9% respectively; p = 0.37). The equal abundance of this bacterial cluster persists into the genus level with similar abundance of most of the genus-like groups in adults and young children; only 1 out of 19 groups were significantly different between young children and adults (Figure 4). *Ruminococcus obeum et rel* is the most predominant genus-like group for both adults and children. It is the only group whose average proportion in total microbiota was more than 10% (11.6% (A) and 13.9% (C); p>0.05) (Figure 4). Similar to Clostridium cluster XIVa, the Clostridium clusters I and XI and uncultured Clostridiales showed equal abundance in adults and young children (Figure 3).

Clostridium cluster IV is the second most abundant phylum-like level (L1) group and was significantly more common in adults than in young children (24% vs. 16% respectively; p = 0.02) (Figure 3). Generally, all L2 groups within Clostridium cluster IV showed either equal or higher abundance in adults (Table 1). Within the Clostridium cluster IV, the average proportion of *F. prausnitzii et rel* in total microbiota was 10.7% (A) and 6.9% (C) (difference not significant) and the abundance of all other groups was below 5%. All bacterial groups had very high variance in the relative abundance between individuals of the same age group and *F. prausnitzii et rel* held the highest variance in young children (data not shown).

Bacteroidetes, the third most common phylum, was significantly more abundant in adults than in children (13% vs. 4.7% respectively; p = 0.02). This is extended to the genus level with a range of 2.4–6.3 higher abundance in adults than in children in eight L2 groups within this phylum (Table 1). All groups had high variance in abundance both in children and adults.

In contrast, Actinobacteria was observed to be the fourth most abundant in both groups, and was significantly less abundant in adults than in children (3.4% vs 12.5% respectively; p<0.01) at the phylum level (Figure 3). This extended to the genus level with *Bifidobacterium* as the only group to show a significant difference (A:3%; C = 11%; p = 0.00008) (Table 1). This held despite the high variance of *Bifidobacterium* abundance between subjects in both the children and adult groups.

Generally, compared to adults, children held a higher abundance of Bacilli and the three predominant groups of this phylum, *Streptococcus bovis*, *S. mitis* and *S. intermedius et rel*, showed significantly higher abundance in children (Table 1). *Enterococcus* had high abundance in some children, but the difference between children and adults was not significant due to high variance.

### III. Diversity of the intestinal microbiota

**Figure 5:**
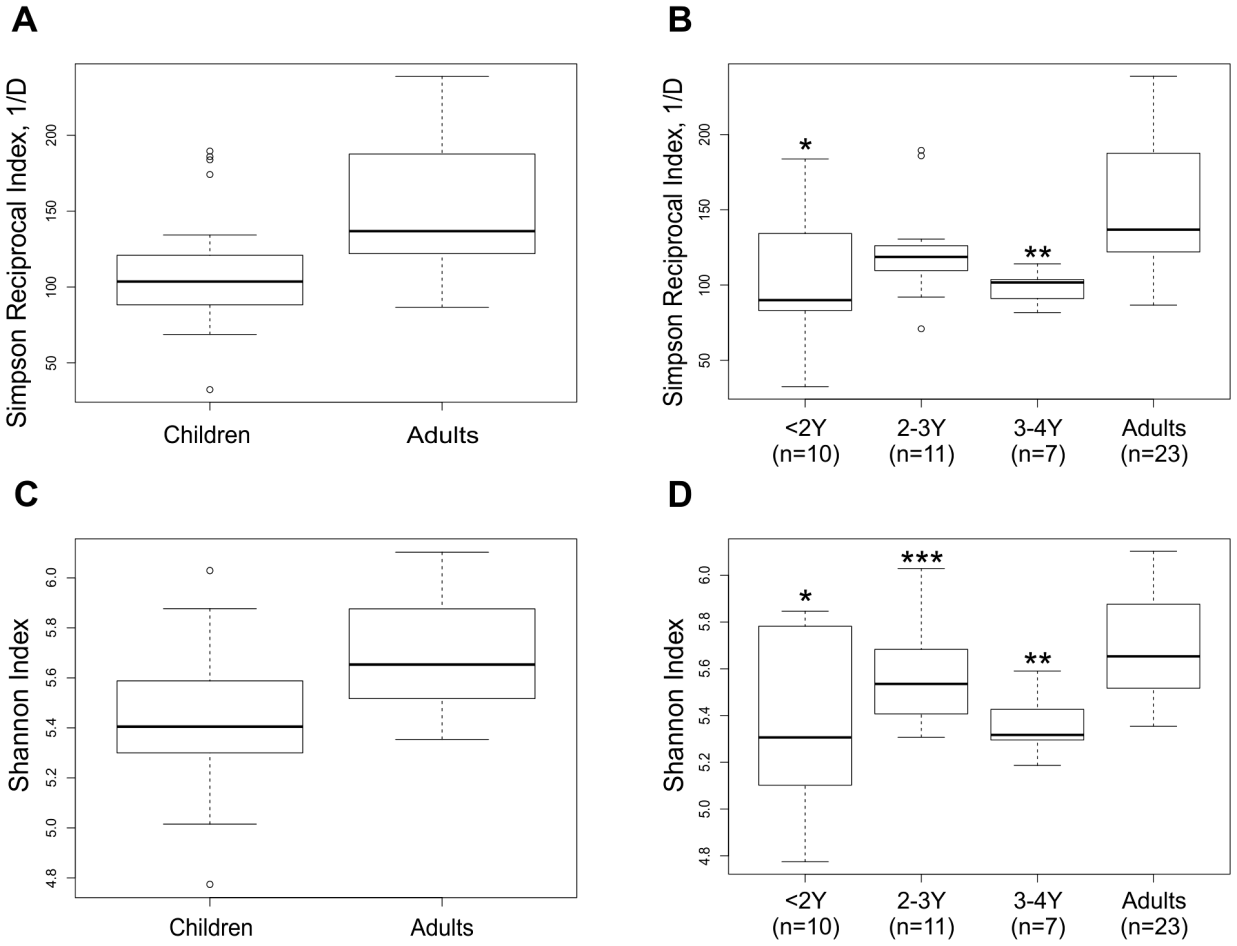
Intestinal microbiota diversity in healthy young children and adults estimated by Simpson reciprocal index (1/D) and Shannon index. The whiskers show the highest and lowest value after excluding outliers (dots). The outliers are defined as more than 3/2 times of upper quartile or less than 3/2 times of lower quartile. Boxplot shows 25^th^ and 75^th^ percentile, with a line at median. The subjects are divided into two groups Adults (>21Y) and Children (<4Y) (A, C) or four groups‘<2Y’, ‘2-3Y’, ‘3-4Y’ and Adults (>21Y) (B, D). A) Simpson index of diversity shows a significant difference between adults and children (p = 0.007), B) Simpson index of diversity shows significant differences between ‘<2Y’and ‘3-4Y’ age groups and adults (*p = 0.006, **p = 0.002 respectively). C) Shannon index of diversity shows significant difference between adults and children (p = 0.001), D) Shannon index of diversity shows significant difference between ‘<2Y’, ‘3-4Y’ and adults, (*p = 0.003, **p = 0.001 respectively). Significant difference is also observed between ‘2-3Y’and ‘3-4Y’ age groups (***p = 0.043).

Generally, the microbiota in adults had significantly higher diversity (abundance and richness) than in young children (Simpson index p = 0.0007, Shannon index p = 0.001) (Figure 5A and 5C respectively). The diversity indices between the age groups of <2 years and 3–4 years were significantly different from adults (Simpson index p = 0.006 and p = 0.002 respectively; Shannon index p = 0.003 and 0.001 respectively) (Figure 5B and 5D, respectively), whereas the diversity between the age group of 2–3 years and adults did not differ. No significant differences in age, gender and race/ethnicity were found in the outliers. Significant difference in diversity was also observed within the children between 2–3 years and 3–4 years age groups (p = 0.043) (Figure 5D). However, lower diversity was evident in most subjects also in this age category as compared to the adults.

### IV. Verification of the most significant microbiota difference by qPCR of Bifidobacterium

**Figure 6:**
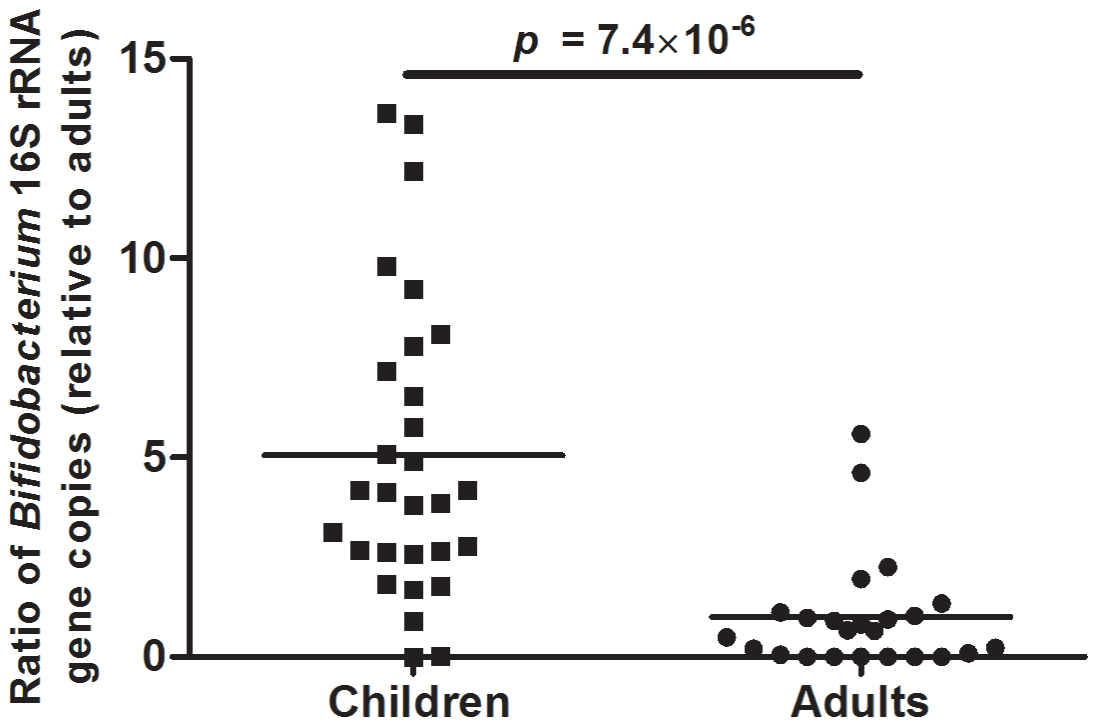
*Bifidobacterium* species adundances in fecal samples from healthy young children and adults reported as a ratio with respect to the average amount of 16S rRNA *Bifidobacterium* gene copies in all adults. A significant 5.05 (±0.70) fold increase was found in healthy young children compared to adults.

qPCR analysis detected *Bifidobacterium* species in all fecal DNA samples. There was a significant 2.7 fold higher abundance of *Bifidobacterium* species in children compared with adults (1.92×10^9^ +/−3.7×10^8^ vs. 7.05×10^8^+/−1.5×10^8^ number of 16S rRNA copies per ug of fecal DNA, respectively; p<0.006) (Figure S5). The levels of *Bifidobacterium* species were 32.1 and 5.0 fold higher in children 1–2 years compared to 2–3 years and 3–4 years, respectively (3.34×10^9^+/−1.04×10^9^ vs. 1.04×10^9^+/−2.03×10^8^; p = 0.015 vs. 1.8×10^9^+/−5.6×10^8^ (Figure S6). This translates into a significant 5.05 fold higher concentration (p = 7.4×10^−6^) of *Bifidobacterium* species in children compared to adult fecal samples (Figure 6). This result is consistent with the microarray data, where higher levels of *Bifidobacterium* were observed in the samples from children (as compared to adults) (Figure 3B and Table 1).

## Discussion

Our study investigates and compares the intestinal microbiota in healthy young children and healthy adults originating from a specific region in North Carolina U.S.A using an advanced high throughput technique investigating the V1 and V6 hypervariable regions of the 16S rRNA gene. By applying highly reproducible phylogenetic microarray analysis we found that although both age groups have considerable microbial compositional similarities there are also significant differences in the diversity of the intestinal microbiota between healthy young children and healthy adults. These findings corroborate those described in a recent comprehensive study with subjects from three continents using a different platform investigating the V4 16S rRNA variable region [32]. Our studyextends this work by focusing on a single geographic region and applying a different approach that provides a different view and additional information as described below.

Our finding of significantly higher diversity (Simpson and Shannon diversity indices) in the abundance and richness of the intestinal microbiota in adults compared to children is consistent with findings from other previously reported studies [31], [32], [40], [50], [51], [52]. Rajilic-Stojanovic et al., found the median of Simpson diversity index of around 150 in healthy adults [52], which is similar to the 136.8 in our healthy adult group. Yatsunenko et al., found that the diversity increased with age when addressing the numbers of OTUs. Our data is in accordance with this finding, and adds an additional measure of diversity by assessing both the richness and evenness of the microbiota through the Simpson reciprocal and Shannon indices, which provide additional confirmation of these findings. We extended the analysis up to 4 years of age and our results suggests that the microbiota has not yet reached the climax of bacterial diversity at this young age. Moreover, by extending the age to 4 years we were able to show significant differences in diversity between the sub-group of 3–4 years and adults. The reasons for lower microbial diversity in children compared to adults are not yet completely understood. It is likely that the accumulation of environmental and dietary exposures over time leads to an increase in diversity with age. Intestinal maturation is likely to affect the mucus production, the glycosylation pattern of mucin glycoproteins and the intestinal immunity, which can have a bearing on the microbiota composition [53], [54], [55], [56]. Support for this hypothesis comes from a recent study looking at a single infant from birth through 2.5 years of age, where an increase in diversity levels in the intestinal microbiota was found as the infant grew [50]. In addition, higher microbial diversity is suggested to be a marker of a healthy intestinal microbiota, as low microbial diversity in adults has been described in association with certain disease conditions such as inflammatory bowel diseases [57] and irritable bowel syndrome [33].

We found separation of the young children population age 1–4 years from adults (21–60 years) using PCA and RDA analyses. The study by Yatsunenko et al., found that children from 0.5 to 2 years cluster either separately or together with adults and that subjects from 2 to 20 years could not be separated from adults over 20 years of age [32]. These differences may be due to the use of different techniques, source population within the U.S., and the different age groups cut-off point (<2 years and 2–20 years). Nevertheless, the clustering results confirm the difference between young children and adults. Our study findings extend this significant difference to 4 years and indicate that the evolvement into adult-like microbiota continues beyond 3 years.

At the phylum-like level, we found that the predominant bacterial groups in children and adults are similar, with Firmicutes, Bacteroidetes, and Actinobacteria being the predominant phyla. These findings are consistent with other studies investigating the intestinal microbiota in adults and children [2], [11], [32], [58], [59] that reported Firmicutes and Bacteroidetes as the major bacterial groups in the human intestinal microbiota, and constitute 60–80% and 15–30% of the total bacteria in adults, respectively [2], [11], [32], [58], [59].

Interestingly, although Clostridium cluster XIVa has been found to be the most abundant bacterial group in both adults and children [59], [60], [61], we did not find a significant difference between young children and adults with respect to this group, suggesting that this phylum group may have already stabilized at an early age. This is further supported by the fact that in the genus level, only 1 out of 19 Clostridium XIVa genera were significantly different between the children and the adult groups. Clostridium XIVa is a major component of the Firmicutes phylum and includes a number of important butyrate-producing bacteria including *Ruminococcus obeum* et rel., which constituted more than 10% of the total microbiota in both adults and children. These bacteria provide the host with butyrate, a short-chain fatty acid, which is a major energy source for enterocytes and supports the mucosal physiology by stimulating motility, mucous secretion, sodium and water absorption and regulating epithelial barrier and immunity functions of the intestinal epithelial cells [62]. The establishment of Clostridium cluster XIVa at an early age highlights its importance in the symbiosis between the human host and the intestinal microbiota.

At the genus-like level, we identified 26 phylogenetic groups that were significantly different between young children and adults and had an abundance higher than 0.01% (Table 1). We found significantly higher (3.7 fold) levels of bifidobacteria in the children group compared to the adult group (K:11%, A:3%, p = 0.00008). Indeed, the *Bifidobacterium* genus, the most abundant genus within Actinobacteria, was the most significant group that lead to the separation of microbiota composition between children and adults in our study. Although higher levels of bifidobacteria in children have been previously reported in numerous European and Japanese studies [63], it was recently suspected that U.S. children may lack significant amounts of bacteria of this group[31]. Our study shows that bifidobacteria are also among the predominant bacteria in U.S. children too. Interestingly, a recent study using a microarray technique in adolescents reported findings in a similar direction with higher (nearly two-fold) levels of *Bifidobacterium* genus in the adolescent group compared to the adult group (9% vs, 5.4% respectively; p<0.01)[64]. This medium level of relative abundance of 9% in adolescents compared to higher (11%) in young children and lower (3% and 5.4%) in adults may represent a gradual decrease in *Bifidobacterium* levels across ages through adulthood.

Breastfed infant have been shown to harbor more bifidobacteria and lactobacilli than formula fed infants [24], [28]. In our study, we did not find a correlation between *Bifidobacterium* levels and history of and duration of breastfeeding (data not shown). However, we were unable to differentiate between children who had received exclusive versus mixed breastfeeding.

Palmer et al. used a microarray technique based on the small subunit ribosomal RNA (SSU rRNA) to investigate the development of fecal microbiota in fourteen infants by frequent sampling until one year of age and comparing to their parents microbiota. This study concluded that colonization of the intestinal microbiota “converged toward a profile characteristic of the adult gastrointestinal tract”, at the age of one year [31]. Our results show that the intestinal microbiota of children up to 4 years of age is different from that of adults suggesting that the colonization to ‘adult-like’ microbiota is not yet complete at one year of age. Thus, our study complements and extends Palmers et al. 's finding in infants up to one year of age. Our data investigating children at 1–4 years suggests that the intestinal microbiota convergence toward a profile characteristic of the adult gastrointestinal tract may occur later than previously expected and may excede at least up to 4 years of age.

Additional strengths of our study are the relatively large number of individuals investigated (children n = 28, adults n = 23), the distinct population from a specific geographical region, the well-defined study population, and the use of strict eligibility criteria to exclude subjects with disease conditions.

The limitations of our study are inherent to one-point study designs and the limited ability to control for the multiple factors that can affect the intestinal microbiota (e.g., diet, exclusive breastfeeding in infancy, siblings, pets, gut physiology and hormone levels). In addition, due to the biases in gender (more women than men), ethnicity (more Caucasian) and BMI category in the adults group (6 out of 23 overweight or obese), we are not able to determine conclusively the effect of these factors on the microbiota of adults. We observed in total eight genus-like level bacterial groups to be affected by either gender, ethnicity or BMI category within the adult group (data not shown). However, none of the eight groups showed difference between children and adults. Therefore, we consider that the differences in microbiota composition between adults and children are more likely due to the age rather than the other confounding factors.

In conclusion, we observed a lack of adult-like microbiota composition in young children. This lack of maturity suggests that the intestinal microbiota may still be vulnerable to external exposures and, on the other hand, may provide an extended window-of-opportunity for interventions aiming to promote health and disrupt disease pathogenesis. While the Clostridium cluster XIVa seems to be established by age 4, earlier than other phyla, Bacteroidetes, *Bifidobacteria* and Clostridium cluster IV are expected to gradually change with age. Additional studies are needed to assess the temporal stability of Clostridium XIVa throughout childhood.

Our findings of compositional changes in the intestinal microbiota in young children, coupled with findings reported by others [32], [64], strengthen the assertion that with additional research using advanced molecular techniques, we will be able to understand better the colonization process through early and late childhood. These studies need to take into account factors such as diet, physiology and other variables which may contribute to the development of the intestinal microbiota through age, as well as functionality analysis that will provide insight into metabolic pathways. Together these will advance our understanding of healthy gut colonization and provide opportunities to improve health and prevent disease conditions through evidence-based interventions aiming to preserve and promote healthy intestinal colonization.

## Supporting Information

Figure S1 Barplot of the demographics of the study population coloured by groups. A) Gender info. B) Race info. C) BMI category. The groups in Adults are clearly unevenly distributed. Body mass index (BMI) was calculated per standard definition (kg/m^2^). For adults, weight and height were collected at their visit and BMI was calculated and characterized as healthy weight (BMI 18.5–24.9), overweight (BMI 25–29.9) and obese (BMI ≥30). For children, weight and height were collected at the child care center by trained research assistants. BMI percentile was determind using the World Health Organization (WHO) age and sex adjusted referencestandards. Applying these percentiles, children were characterized as healthy (5–84.9%), overweight (85%–94.9%) or obese (≥95%).
(PDF)

Figure S2 Principal component analysis (PCA) (panels A, C, E and G) and redundancy analysis (RDA) (panels B, D, F and H) of fecal samples from healthy young children at the genus-like level showing gender, ethnicity, BMI category and age group of the subjects. Log transformed data were used for analysis. In PCA, the first two principal components capture 29% (PCA1) and 17% (PCA2) of variance respectively. RDA plot shows the result from supervised PCA, where group assignment of subjects (gender, ethnicity, BMI category or age group) was used as a dependent variable. The ethnicity group “other” includes 3 hispanic, 1 pacific-islander and 1 child, whose ethnicity was not recorded. In RDA, first and second ordination axes are plotted and the proportion of variance explained (%) is indicated. p value obtained by permutation test is reported (p values below 0.05 are considered significant).
(PDF)

Figure S3 Principal component analysis (PCA) (panels A, C and E) and redundancy analysis (RDA) (panels B, D and F) of fecal samples from healthy adults at the genus-like level showing gender, ethnicity and BMI category of the subjects. Log transformed data were used for analysis. In PCA, the first two principal components capture 37% (PCA1) and 12% (PCA2) of variance respectively. RDA plot shows the result from supervised PCA, where group assignment of subjects (gender, ethnicity or BMI category) was used as a dependent variable. The ethnicity of the “other” is Asian. In RDA, first and second ordination axes are plotted and the proportion of variance explained (%) is indicated. p value obtained by permutation test is reported (p values below 0.05 are considered significant).
(PDF)

Figure S4 Principal component analysis (PCA) (panels A, C and E) and redundancy analysis (RDA) (panels B, D and F) of fecal samples from healthy young children and adults at the phulym-like level showing gender, ethnicity, BMI category and age group of the subjects. Log transformed data were used for analysis. In PCA, the first two principal components capture 21% (PCA1) and 16% (PCA2) of variance respectively. RDA plot shows the result from supervised PCA, where group assignment of subjects (gender, ethnicity, BMI category or age group) was used as a dependent variable. The ethnicity group “other” includes 1 asian adult, 3 hispanic children, 1 pacific-islander child and 1 child, whose ethnicity was not recorded. In RDA, first and second ordination axes are plotted and the proportion of variance explained (%) is indicated. p value obtained by permutation test is reported (p values below 0.05 are considered significant).
(PDF)

Figure S5 Levels of *Bifidobacterium* species in Fecal Samples from Healthy Young Children and Adults. *Bifidobacterium* species are expressed as the number of 16S rRNA copies per ug of fecal DNA. The levels of *Bifidobacterium* species are 2.7 folder higher in kid samples when compared to adult samples.
(TIF)

Figure S6 Levels of *Bifidobacterium* species in Fecal Samples from Healthy Young Children Stratified by Age. *Bifidobacterium* species are expressed as the number of 16S rRNA copies per ug of fecal DNA. The levels of *Bifidobacterium* species are 32.1 and 5.0 folder higher in kids aged 1–2 years when compared to kids aged 2–3 and 3–4 years, respectively.
(TIF)

Table S1 Demographics Information of Study Subjects
(XLSX)

Table S2 HITChip Raw Data.
(PDF)
