# Supplementary figures and images for: Intestinal Microbiota in Healthy U.S. Young Children and Adults—A High Throughput Microarray Analysis
Source: PLoS One. 2013 May 23;8(5):e64315. doi: 10.1371/journal.pone.0064315 (PMC3662718; doi:10.1371/journal.pone.0064315)

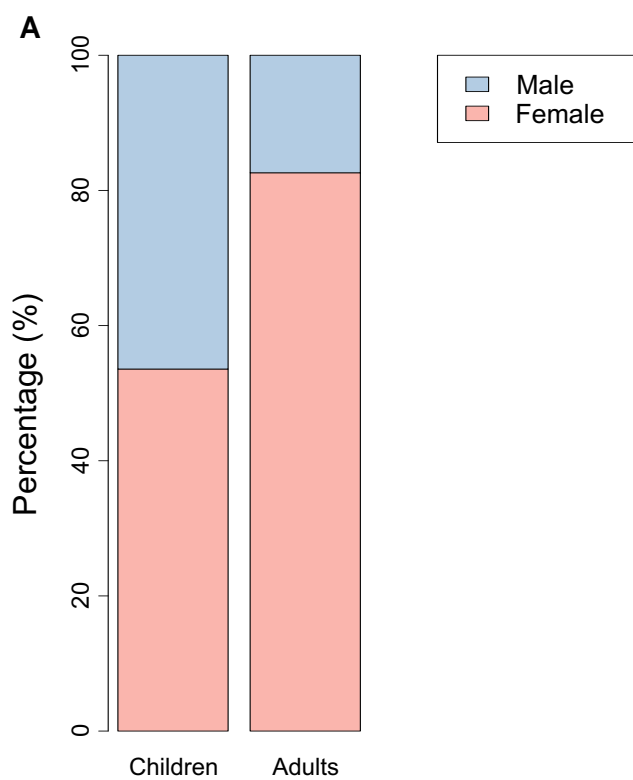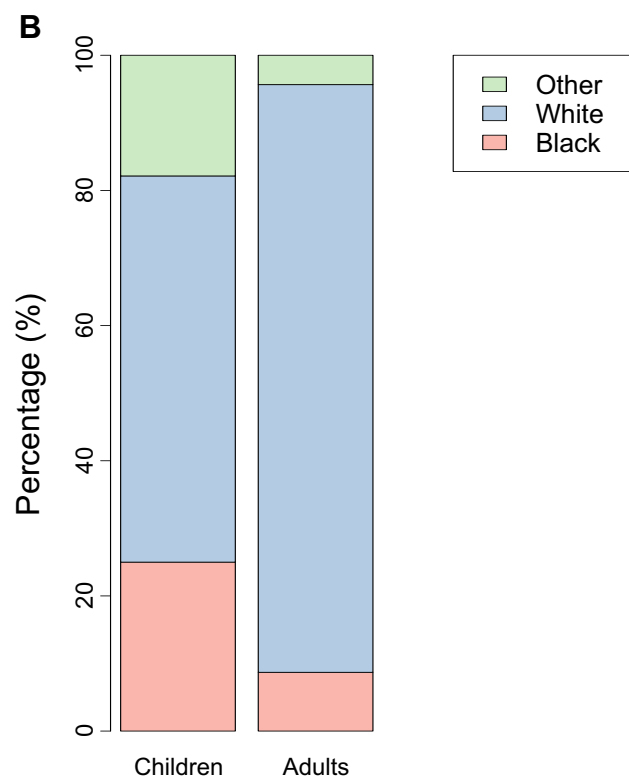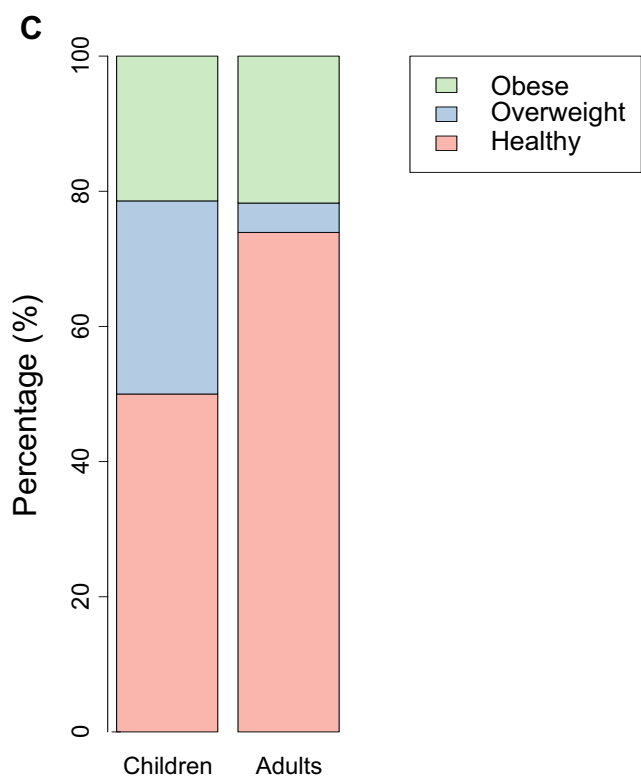

Supplement: Figure S1 — Barplot of the demographics of the study population coloured by groups. A) Gender info. B) Race info. C) BMI category. The groups in Adults are clearly unevenly distributed. Body mass index (BMI) was calculated per standard definition (kg/m2). For adults, weight and height were collected at their visit and BMI was calculated and characterized as healthy weight (BMI 18.5–24.9), overweight (BMI 25–29.9) and obese (BMI ≥30). For children, weight and height were collected at the child care center by trained research assistants. BMI percentile was determind using the World Health Organization (WHO) age and sex adjusted referencestandards. Applying these percentiles, children were characterized as healthy (5–84.9%), overweight (85%–94.9%) or obese (≥95%). (PDF) [file pone.0064315.s001.pdf]

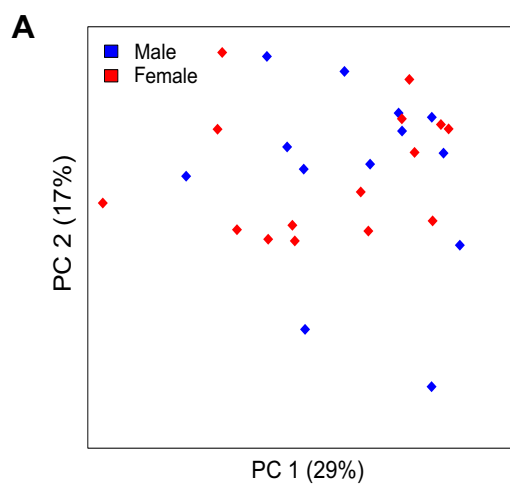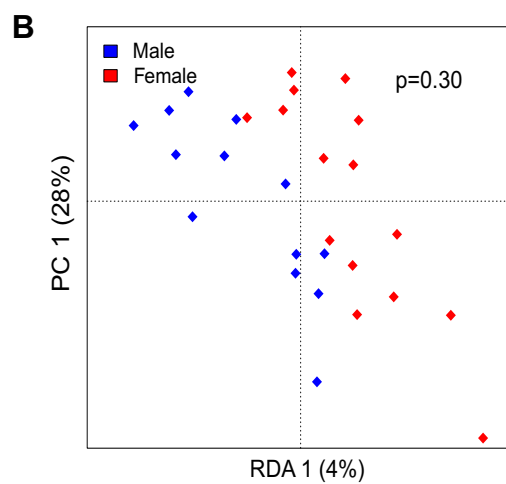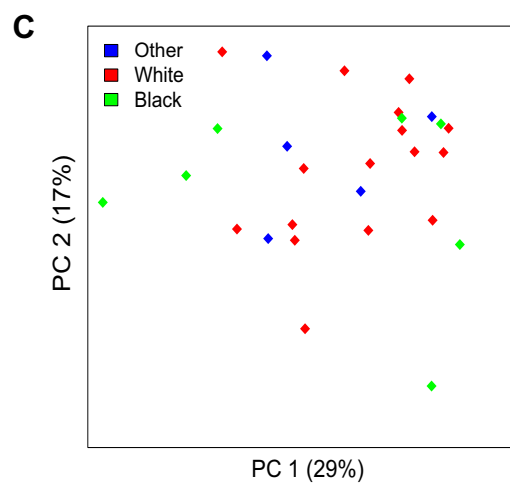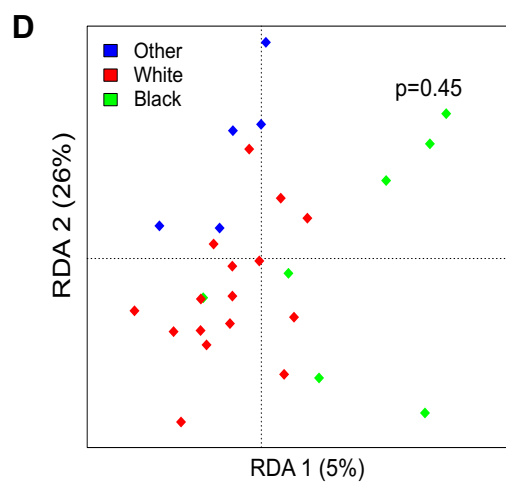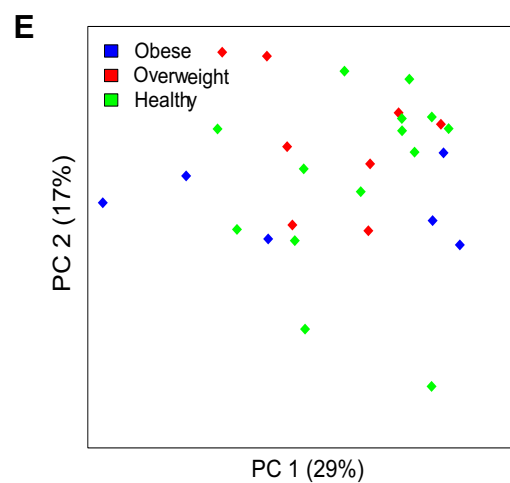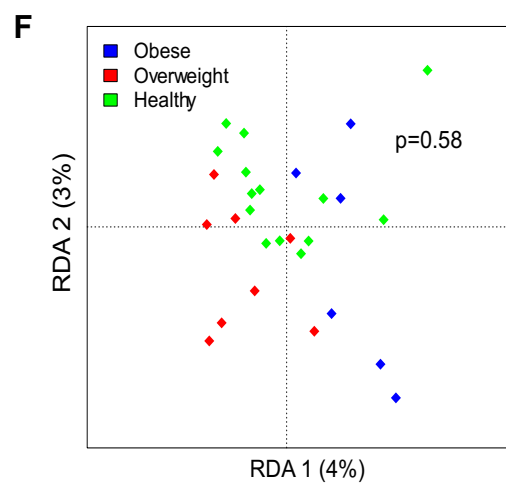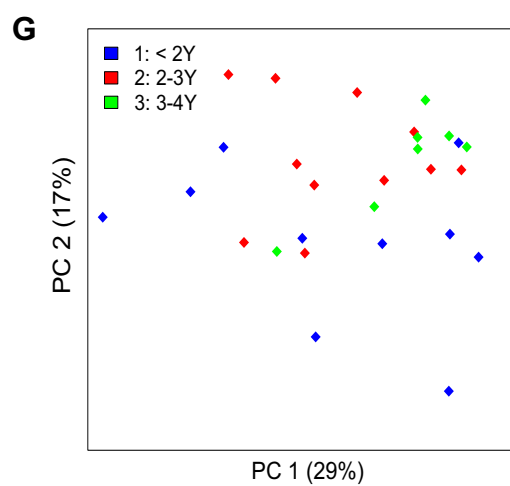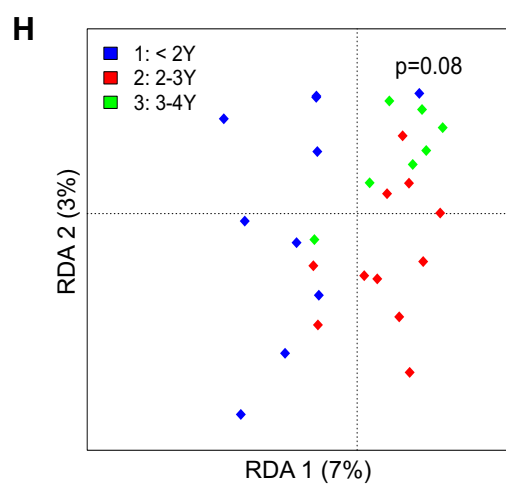

Supplement: Figure S2 — Principal component analysis (PCA) (panels A, C, E and G) and redundancy analysis (RDA) (panels B, D, F and H) of fecal samples from healthy young children at the genus-like level showing gender, ethnicity, BMI category and age group of the subjects. Log transformed data were used for analysis. In PCA, the first two principal components capture 29% (PCA1) and 17% (PCA2) of variance respectively. RDA plot shows the result from supervised PCA, where group assignment of subjects (gender, ethnicity, BMI category or age group) was used as a dependent variable. The ethnicity group “other” includes 3 hispanic, 1 pacific-islander and 1 child, whose ethnicity was not recorded. In RDA, first and second ordination axes are plotted and the proportion of variance explained (%) is indicated. p value obtained by permutation test is reported (p values below 0.05 are considered significant). (PDF) [file pone.0064315.s002.pdf]

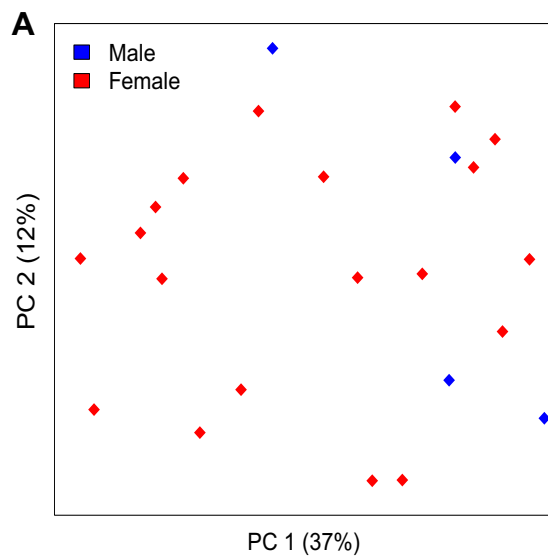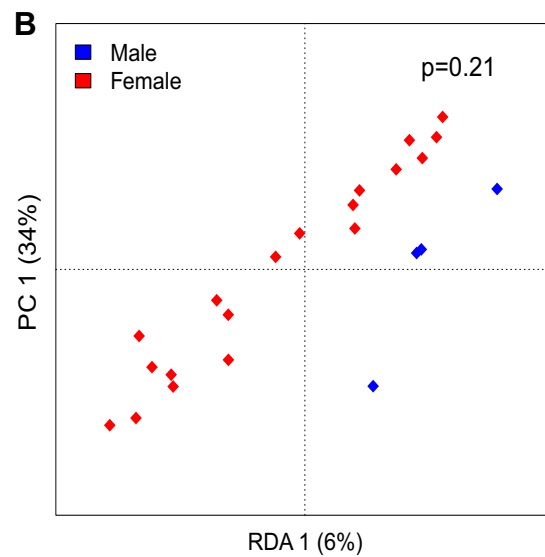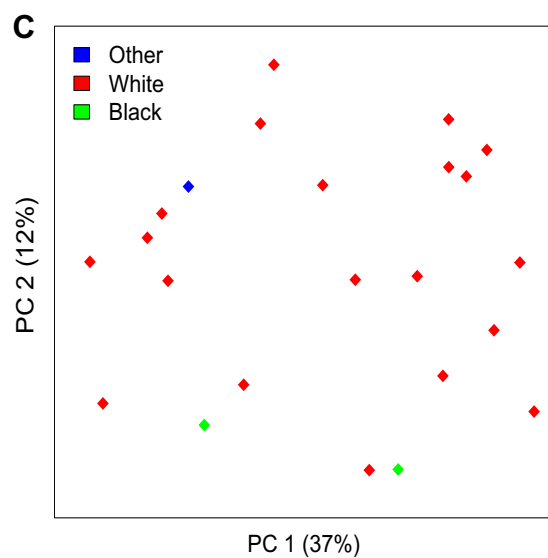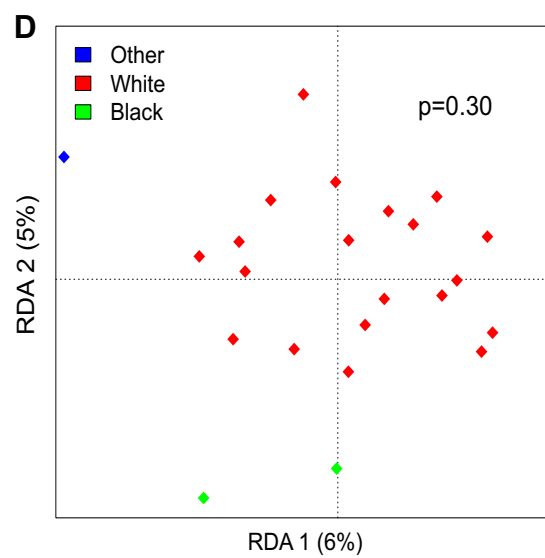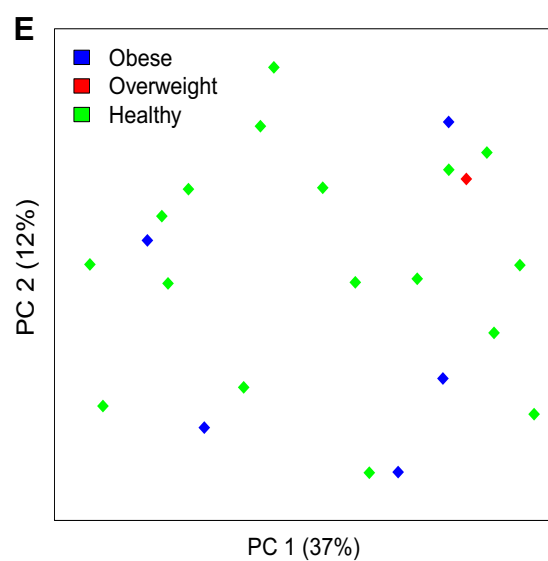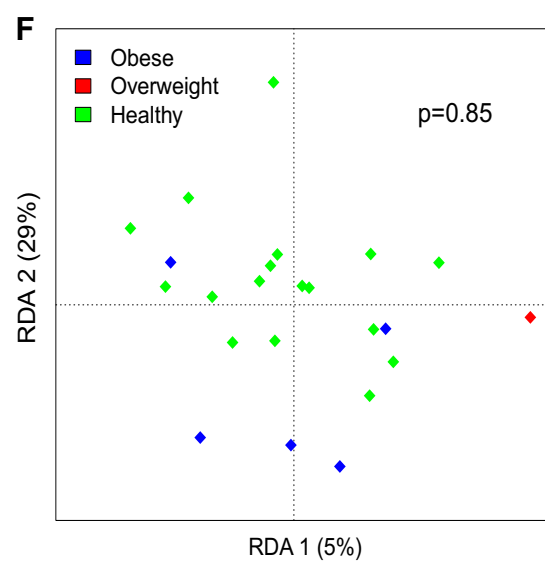

Supplement: Figure S3 — Principal component analysis (PCA) (panels A, C and E) and redundancy analysis (RDA) (panels B, D and F) of fecal samples from healthy adults at the genus-like level showing gender, ethnicity and BMI category of the subjects. Log transformed data were used for analysis. In PCA, the first two principal components capture 37% (PCA1) and 12% (PCA2) of variance respectively. RDA plot shows the result from supervised PCA, where group assignment of subjects (gender, ethnicity or BMI category) was used as a dependent variable. The ethnicity of the “other” is Asian. In RDA, first and second ordination axes are plotted and the proportion of variance explained (%) is indicated. p value obtained by permutation test is reported (p values below 0.05 are considered significant). (PDF) [file pone.0064315.s003.pdf]

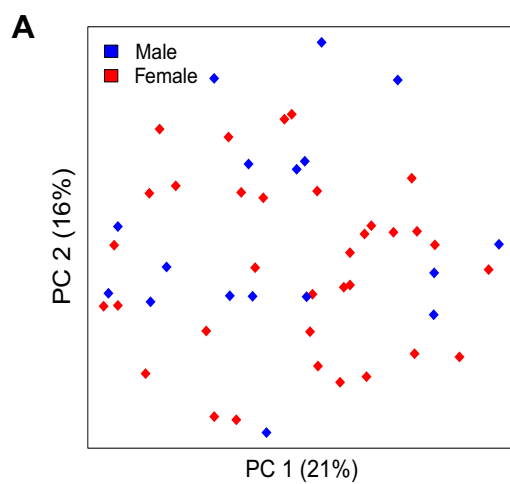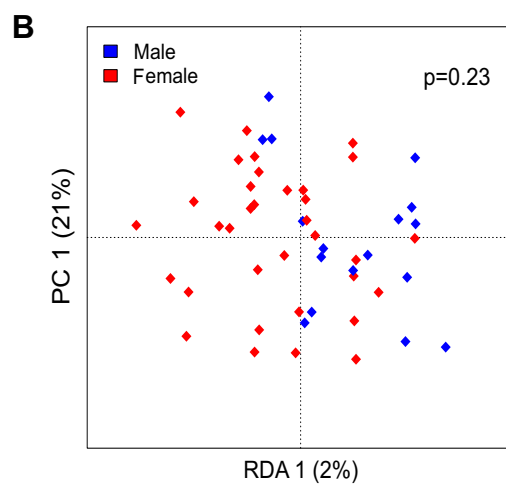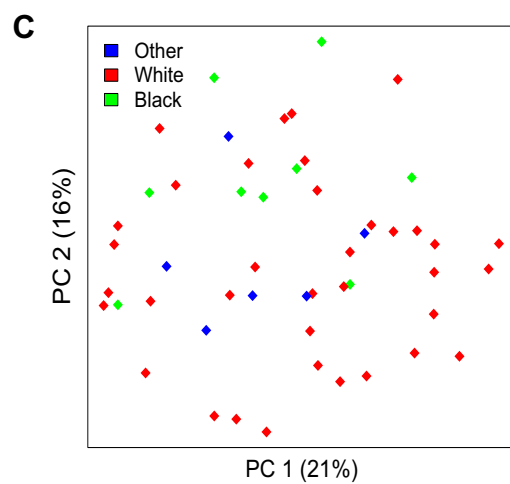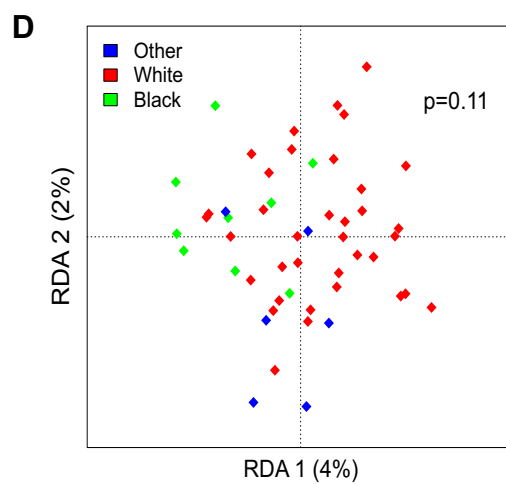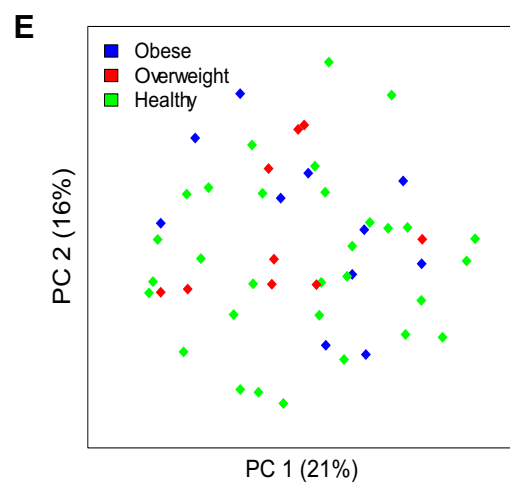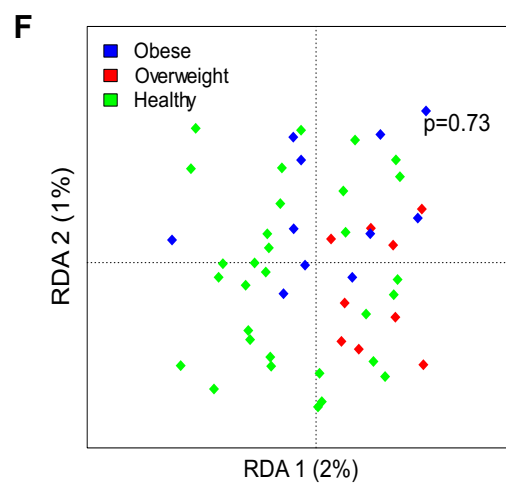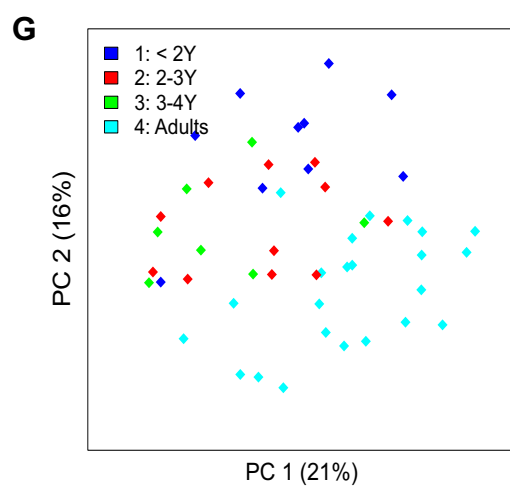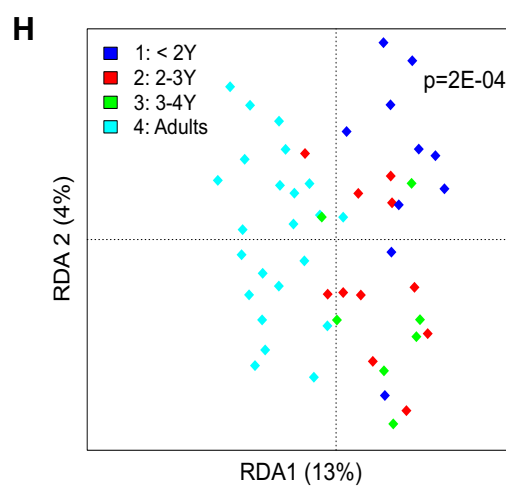

Supplement: Figure S4 — Principal component analysis (PCA) (panels A, C and E) and redundancy analysis (RDA) (panels B, D and F) of fecal samples from healthy young children and adults at the phulym-like level showing gender, ethnicity, BMI category and age group of the subjects. Log transformed data were used for analysis. In PCA, the first two principal components capture 21% (PCA1) and 16% (PCA2) of variance respectively. RDA plot shows the result from supervised PCA, where group assignment of subjects (gender, ethnicity, BMI category or age group) was used as a dependent variable. The ethnicity group “other” includes 1 asian adult, 3 hispanic children, 1 pacific-islander child and 1 child, whose ethnicity was not recorded. In RDA, first and second ordination axes are plotted and the proportion of variance explained (%) is indicated. p value obtained by permutation test is reported (p values below 0.05 are considered significant). (PDF) [file pone.0064315.s004.pdf]

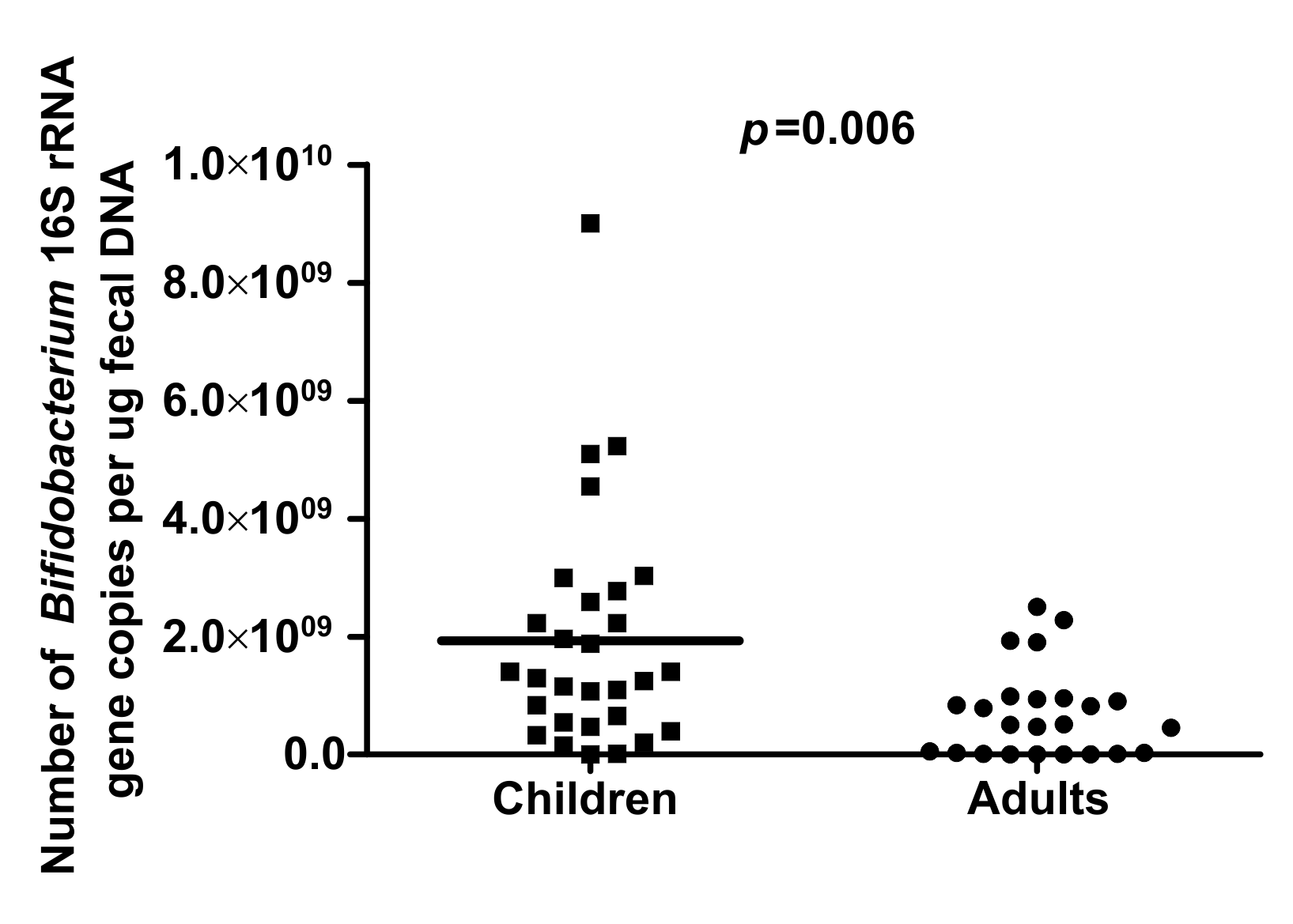

Supplement: Figure S5 — Levels of Bifidobacterium species in Fecal Samples from Healthy Young Children and Adults. Bifidobacterium species are expressed as the number of 16S rRNA copies per ug of fecal DNA. The levels of Bifidobacterium species are 2.7 folder higher in kid samples when compared to adult samples. (TIF) [file pone.0064315.s005.tif]

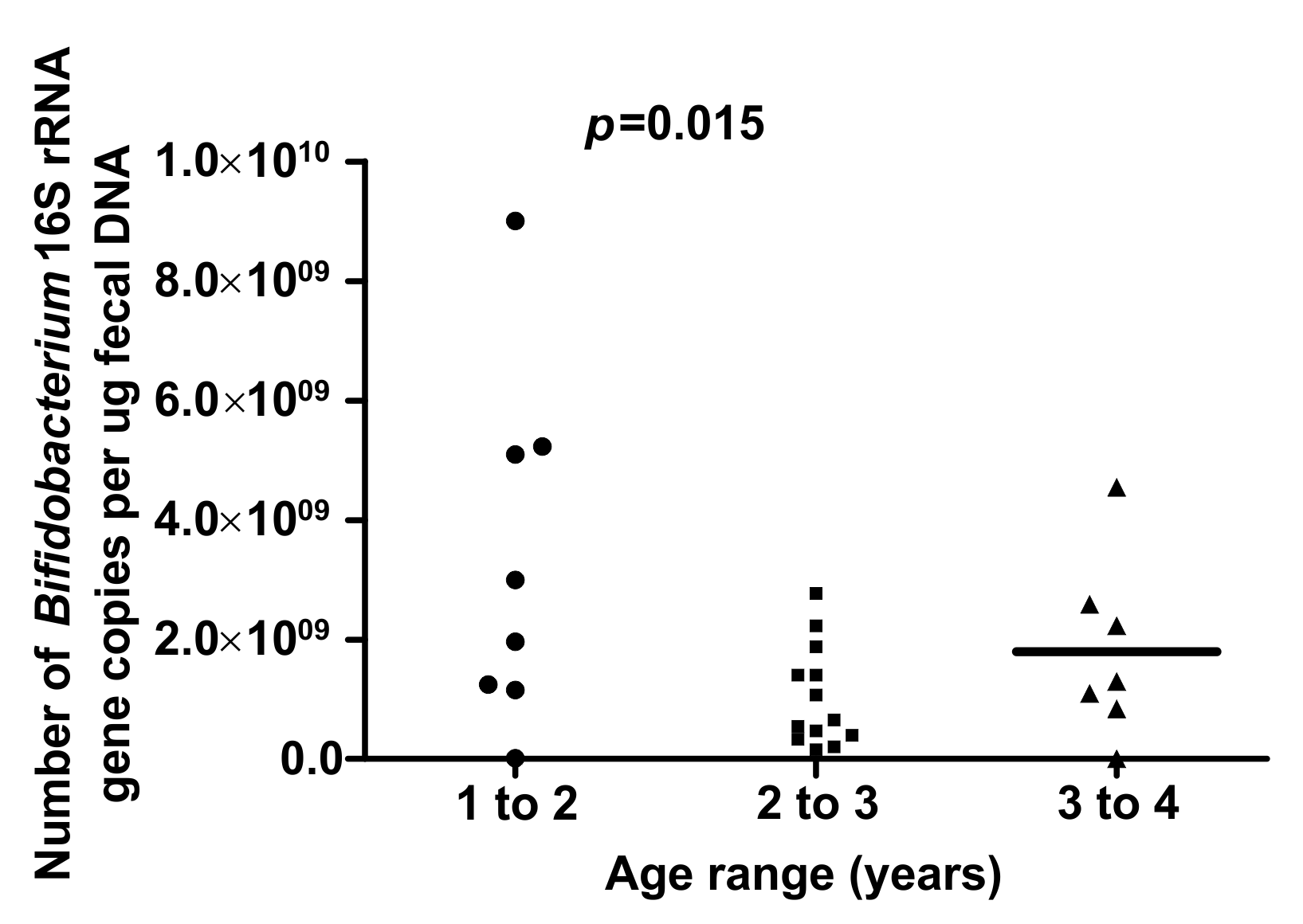

Supplement: Figure S6 — Levels of Bifidobacterium species in Fecal Samples from Healthy Young Children Stratified by Age. Bifidobacterium species are expressed as the number of 16S rRNA copies per ug of fecal DNA. The levels of Bifidobacterium species are 32.1 and 5.0 folder higher in kids aged 1–2 years when compared to kids aged 2–3 and 3–4 years, respectively. (TIF) [file pone.0064315.s006.tif]
